# Chemical Dissection of PM_2.5_ in Cigarette Smoke: Main and Sidestream Emission Factors and Compositions

**DOI:** 10.3390/toxics13090711

**Published:** 2025-08-23

**Authors:** Yujian Zhou, Hong Huang, Changwei Zou, Mengmeng Deng, Xiang Tu, Wei Deng, Chenglong Yu, Jianlong Li

**Affiliations:** 1 School of Resources & Environment, Nanchang University, Nanchang 330031, China; 412300230077@email.ncu.edu.cn (Y.Z.); cwzou@ncu.edu.cn (C.Z.); 352300220005@email.ncu.edu.cn (M.D.); jlli@ncu.edu.cn (J.L.); 2 Jiangxi Provincial Key Laboratory of Environmental Pollution Control, Jiangxi Academy of Ecological Environment Science and Planning, Nanchang 330039, China; tuxiang@sthjt.jiangxi.gov.cn; 3 School of Land Resources and Environment, Jiangxi Agricultural University, Nanchang 330045, China; hjclyu@jxau.edu.cn

**Keywords:** main and sidestream smoke of cigarette, PM_2.5_, chemical compositions, emission factors

## Abstract

Despite increasing evidence that cigarette smoke is a significant source of indoor fine particulate matter (PM_2.5_), quantitative emission factors (EFs) for PM_2.5_ and its toxic chemical composition in mainstream (MS) and sidestream (SS) smoke are still not well defined. In this study, we employed a custom-designed chamber to separately collect MS (intermittent puff) and SS (continuous sampling) smoke from eleven cigarette models, representing six brands and two product types, under controlled conditions. PM_2.5_ was collected on quartz-fiber filters and analyzed for carbon fractions (using the thermal–optical IMPROVE-A protocol), nine water-soluble inorganic ions (by ion chromatography), and twelve trace elements (via ICP-MS). SS smoke exhibited significantly higher mass fractions of total analyzed species (84.7% vs. 65.9%), carbon components (50.6% vs. 44.2%), water-soluble ions (17.1% vs. 13.7%), and elements (17.0% vs. 7.0%) compared to MS smoke. MS smoke is characterized by a high proportion of pyrolytic organic carbon fractions (OC_1_–OC_3_) and specific elements such as vanadium (V) and arsenic (As), while SS smoke shows elevated levels of elemental carbon (EC1), water-soluble ions (NH_4_^+^, NO_3_^−^), and certain elements like zinc (Zn) and cadmium (Cd). The toxicity-weighted distribution indicates that MS smoke primarily induces membrane disruption and pulmonary inflammation through semi-volatile organics and elements, whereas SS smoke enhances oxidative stress and cardiopulmonary impairment via EC-mediated reactions and secondary aerosol formation. The mean OC/EC ratio of 132.4 in SS smoke is an order of magnitude higher than values reported for biomass or fossil-fuel combustion, indicative of extensive incomplete combustion unique to cigarettes and suggesting a high potential for oxidative stress generation. Emission factors (µg/g cigarette) revealed marked differences: MS delivered higher absolute EFs for PM_2.5_ (422.1), OC (8.8), EC (5.0), Na^+^ (32.6), and V (29.2), while SS emitted greater proportions of NH_4_^+^, NO_3_^−^, Cl^−^, and carcinogenic metals (As, Cd, Zn). These findings provide quantitative source profiles suitable for receptor-oriented indoor source-apportionment models and offer toxicological evidence to support the prioritization of comprehensive smoke-free regulations.

## 1. Introduction

Indoor air pollution from cigarette smoke remains a leading yet modifiable risk factor for global morbidity and mortality. Worldwide, 1.30 billion current smokers generate an estimated 209 million kg of PM_2.5_ annually, of which >80% is released indoors and contributes 25–45% to total indoor PM_2.5_ in residences, vehicles, and hospitality venues [1]. Meta-analysis of 42 epidemiological cohorts links every 10 µg m^−3^ increment in cigarette-derived PM_2.5_ to a 12% rise in all-cause mortality (95% CI: 1.08–1.16), a 17% increase in asthma hospitalization among children, and a 14% increase in low-birth-weight deliveries among pregnant non-smokers [2]. Active smoking causes >8 million deaths yr^−1^, whereas secondhand smoke (SHS) accounts for an additional 1.3 million deaths yr^−1^, with 28% of these attributed exclusively to fine particle exposure [3]. These figures underscore the urgency of precisely characterizing the emission properties of both mainstream (MS) and sidestream (SS) smoke.

Cigarette smoke constitutes a significant source of PM_2.5_ emissions in both indoor and outdoor environments, with particularly severe impacts in enclosed spaces. Smoke-derived particulate matter is predominantly composed of fine particles, with most particles measuring less than 2.5 μm [4]. These emissions present a triple threat, contributing to environmental pollution, climate effects, and substantial health risks. Numerous studies have demonstrated that both direct smoking and secondhand exposure significantly increase the incidence of respiratory diseases, cardiovascular disorders, and various cancers [5,6,7], thereby endangering both smokers and non-smokers. To properly evaluate these multifaceted impacts, researchers must conduct comprehensive analyses of cigarette smoke emissions. This requires detailed characterization of particulate matter, including its chemical composition, size distribution, and emission patterns. Of particular importance is quantifying the emission factors and determining the physicochemical properties of PM_2.5_ from both MS and SS smoke produced by modern cigarettes, which increasingly incorporate advanced tobacco processing technologies.

Many research has established that MS cigarette smoke poses significant health risks to smokers [8,9,10]. While MS smoke contains various gaseous pollutants—including carbon monoxide, formaldehyde, hydrogen cyanide, volatile organic compounds, ammonia, hydrogen sulfide, and polycyclic aromatic hydrocarbons—the particulate matter (PM) component, particularly fine particles, presents greater health hazards [11]. These fine particles can penetrate deeper into the respiratory system, posing direct health threats to smokers [4].

SS smoke, emitted from the burning end of cigarettes, contains distinct PM emission characteristics compared to MS smoke [12] and affects both smokers and nearby non-smokers [13]. Notably, studies indicate that PM_2.5_ from SS smoke may pose greater health risks to vulnerable populations, including children and pregnant women, than mainstream smoke exposure [14].

Despite these findings, current research lacks systematic comparative analyses of PM_2.5_ chemical composition between MS and SS cigarette smoke—a critical knowledge gap that hinders accurate exposure assessment and health risk evaluation. Our study addresses this gap through comprehensive chemical characterization of PM_2.5_, quantifying emission factors for toxic trace elements (As, Cd, Cr, Ni, etc.), water-soluble ions (NH_4_^+^, Cl^−^, NO_3_^−^, etc.) and carbonaceous components (OC and EC) across representative cigarette brands. Using a chemical dissection approach, this study aims to (1) establish source-specific emission profiles to accurately differentiate MS and SS smoke characteristics in indoor environments; (2) evaluate how product characteristics (filter design, cigarette diameter) mechanistically influence particulate emissions; and (3) provide scientific evidence supporting smoke-free policies by demonstrating SS smoke’s predominant contribution to indoor oxidative stress and persistent particulate pollution. These findings offer crucial data for protecting vulnerable populations from secondhand smoke exposure while establishing an innovative methodological framework for tobacco smoke characterization research.

## 2. Materials and Methods

### 2.1. Cigarettes Selected for the Experiment

For this investigation, we selected eleven cigarette models representing six distinct brands, encompassing two major cigarette varieties: flue-cured tobacco and popper filter types. Flue-cured (FC) cigarettes are manufactured solely from flue-cured Virginia tobacco dried at 60–70 °C in bulk curing barns. The high reducing-sugar content of this leaf promotes the formation of semi-volatile carbonyls and sugar-dehydration products. Capsule-filter (CF) cigarettes featured a distinctive 3–5 mm breakable capsule embedded in the cellulose-acetate filter containing 10–15 μL of flavoring agents such as menthol or fruit esters. The detailed material parameters of these selected cigarette samples are systematically presented in Table S1.

### 2.2. Collection of PM_2.5_ Sample from Main and Sidestream Smoke of Cigarettes

A custom-designed transparent plastic chamber served as the experimental enclosure equipped with operating gloves and was predominantly sealed to maintain a controlled environment. The chamber’s internal dimensions were 0.85 m (length) × 0.75 m (width) × 0.60 m (height), with a volume of 0.3825 m^3^. The sampler in the chamber was operated manually.

**Figure 1:**
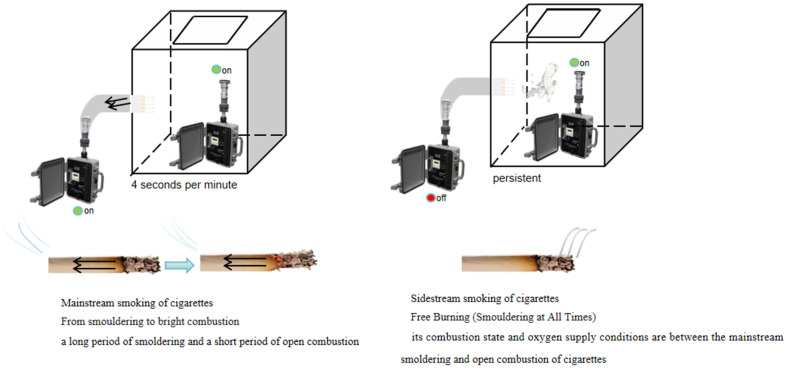
Schematic diagram of PM_2.5_ sampling in MS and SS smoke of cigarettes by chamber.

The method and steps for collecting PM_2.5_ from MS smoke are as follows. Five cigarettes were examined concurrently within a single experimental run. The parallel configuration serves two purposes: to attenuate run-to-run variability arising from ambient fluctuations, and to derive an ensemble-averaged emission factor that more faithfully reflects the product-level mean. Each cigarette constituted an independent replicate; the cigarettes were individually secured in five discrete apertures of the exposure chamber, oriented such that the filter tip protruded externally while the combustion end remained within the enclosure. AirMetrics MiniVol^®^ Tactical Air Sampler (TAS; AirMetrics, Springfield, OR, USA) outside the chamber was connected to the filter tips of cigarette via reducer pipe, with a rubber tube linking the sampler to the mouthpiece. The complete experimental arrangement is depicted in Figure 1, which schematically illustrates the parallel sampling of both MS and SS PM_2.5_ within the exposure chamber. The interface was sealed with tape to prevent the escape of MS smoke from the cigarettes. The operator reaches into the chamber with matching rubber gloves and lights the five cigarettes in the chamber, each ignited with a 0.2 s interval, approximating simultaneous ignition. After the cigarette is lit, turn on the pump of the sampler, use the sampler to continue sampling for 4 s, then turn off the pump of the sampler and stop for 6 s, simulating the state and duration of smoking in humans, and continue sampling for another 4 s and stop for 6 s, and so on. The continuous sampling for 4 s and the stop for 6 s is the situation selected in accordance with the “Definition Standards and Conditions for Smoking Machines for Routine Analysis” in China [15], and the purpose of intermittent sampling is to simulate the situation of smokers inhaling MS smoke during manual smoking. This method simulated human smoking behavior, with a smoke inhalation volume of approximately 1 L/min. PM_2.5_ from cigarette MS smoke was collected using a quartz fiber filter membrane (Whatman, 47 mm in diameter Boston, MA, USA). The filter membranes loaded PM_2.5_ samples were preserved at −20 °C for analysis.

The method and steps for collecting PM_2.5_ from SS smoke are as follows. PM_2.5_ in the chamber was collected by another MiniVol TAS concurrently. After the experimental cigarettes were lit, PM_2.5_ was continuously collected in the experimental chamber. Generally speaking, the duration of smoking a cigarette is about 5–10 min. In this study, the continuous collection duration of PM_2.5_ from SS smoke of cigarettes was 5 min for each type of cigarette selected for experiment, respectively.

### 2.3. Determination of PM_2.5_ and Its Chemical Composition

#### 2.3.1. Determination of PM_2.5_ Mass

The mass of PM_2.5_ in MS and SS smoke collected on quartz filter membranes was determined by gravimetric methods.

#### 2.3.2. Determination of Carbon Components

Organic carbon (OC) and elemental carbon (EC) on the 47 mm quartz-fiber filters were quantified with a DRI-2015 multi-band thermal/optical carbon analyzer (Atmoslytic Inc., Calabasas, CA, USA), operated strictly following the IMPROVE-A temperature protocol. The protocol volatilizes OC in four helium steps (140, 280, 480, and 580 °C) and combusts EC in three oxygen steps (580, 740 and 840 °C); laser transmittance at 633 nm is monitored throughout to correct for charring. OC is reported as the sum of OC1–OC4 plus optically determined pyrolyzed carbon (OPC), whereas EC is calculated as (EC1 + EC2 + EC3) − OPC [16].

#### 2.3.3. Determination of Water-Soluble Inorganic Ions (WSIIs)

A quarter of the loaded sample filter membrane was excised using ceramic scissors and fragmented into a 50 mL sterile centrifuge tube. Subsequently, 30 mL distilled water was introduced, and the centrifuge tube was positioned on a test tube rack before being transported to a KQ-300DE CNC ultrasonic instrument to promote the dissolution of water-soluble ionic substances using ultrasound. After ultrasonic extraction, the aqueous extract was transferred to a clean centrifuge tube using a disposable syringe fitted with a 0.22 µm filter needle, and then stored at −4 °C for analysis.

The quantification of water-soluble inorganic cations (Na^+^, NH_4_^+^, K^+^, Mg^2+^, Ca^2+^) was conducted using an ICS-1100 ion chromatograph (Dionex Inc., Sunnyvale, CA, USA) equipped with a CS12A column, a CG12A guard column, an ASRS-300 suppressor, and a 20 mmol/L methane sulfonic acid at a flow rate of 1.0 mL/min.

The quantification of water-soluble inorganic anions (Cl^−^, NO_2_^−^, NO_3_^−^, SO_4_^2−^) was conducted using an ICS-5000 + SP (Dionex Inc., Sunnyvale, CA, USA) ion chromatograph equipped with an AS18 column, an AG18 guard column, an ASRS-300 suppressor, and a 40 mmol/L NaOH aqueous eluent at a flow rate of 1.0 mL/min.

#### 2.3.4. Determination of Elements

Following the National Environmental Protection Standard of China [17] for elemental determination in particulate matter, we processed the collected samples as follows. First, half of each loaded filter membrane was carefully excised using ceramic scissors, shredded into fragments, and transferred to a digestion vessel. We then added 10 mL of a 3:1 (*v*/*v*) HCl-HNO_3_ mixed acid solution to completely immerse the membrane fragments. After covering the vessels, we performed microwave-assisted acid digestion using a programmed heating protocol: 2 h of heating and refluxing at 100 °C, followed by cooling and a 30 min extraction period. The resulting digestate was filtered into a 50 mL volumetric flask and diluted to volume with purified water prior to analysis.

Elemental quantification (As, Ba, Cd, Co, Cr, Cu, Mn, Ni, Pb, Sb, V, and Zn) was performed using high-resolution inductively coupled plasma mass spectrometry (ICP-MS; Prodigy XP-High Dispersion, Teledyne Leeman Labs, Hudson, NH, USA).

#### 2.3.5. Calculation of Emission Factors (EFs)

The emission factor for PM_2.5_ and its chemical composition in the MS and SS cigarette smoke is calculated using Equation (1)

(1) $$A=\frac{\rho \times V}{m}$$

where *A* is the emission factor, μg/g; *ρ* is the mass concentration of PM_2.5_ or its chemical components, μg/m^3^; *m* is the mass of cigarette combustion, g; and *V* is the sampling volume, m^3^.

## 3. Results and Discussion

### 3.1. Distribution of PM_2.5_ and Its Chemical Composition in Smoke of Cigarettes

#### 3.1.1. Percentage Composition of Chemical Components in PM_2.5_

**Figure 2:**
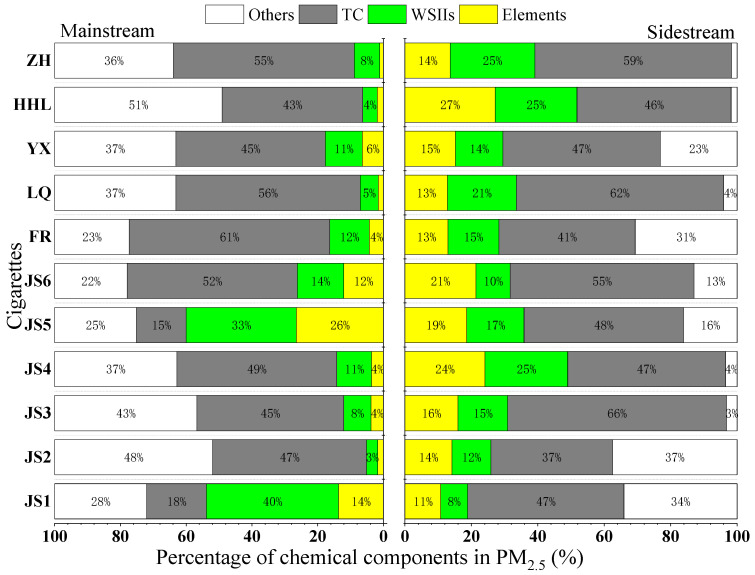
Percentage of chemical components in PM_2.5_ from main and sidestream smoke of cigarettes.

Figure 2 shows the total percentage of total carbon (TC), water-soluble inorganic ions (WSIIs) and trace elements in PM_2.5_ in MS smoke of 11 cigarettes, ranging from 49.1% to 77.9%, with an average of 65.9%. Among these, TC constituted the dominant fraction (18.2–61.02%, mean: 44.15%), followed by WSIIs (4.4–40.2%, mean: 13.7%) and trace elements (1.2–26.5%, mean: 7.0%). In contrast, SS smoke exhibited significantly higher proportions of these composition, collectively representing 62.5–98.3% (mean: 84.7%) of PM_2.5_ mass. TC remained the predominant constituent (36.6–62.3%, mean: 50.6%), while WSIIs (8.1–25.5%, mean: 17.1%) and elements (10.8–27.1%, mean: 17.0%) showed elevated contributions compared to MS smoke.

The particles in MS smoke mainly come from the alternating process of smoldering and burning of the tobacco layer inside the tail burning layer. Among them, the chemical components of PM_2.5_ can directly penetrate into the lungs of smokers. In contrast, PM_2.5_ in SS smoke mainly comes from the free burning outside the tail burning layer. These particles are directly emitted into the surrounding environment, posing a threat to the health of both smokers and non-smokers. The difference in the distribution of chemical components between MS and SS cigarettes is mainly attributed to the following factors.

During MS inhalation, the peripheral region of the cigarette tip undergoes luminous, oxygen-rich combustion with a high temperature (about 800–900 °C), while the interior experiences oxygen-limited smoldering pyrolysis. At this time, organic matter in PM_2.5_ (such as tar, polycyclic aromatic hydrocarbons, etc.) can be fully oxidized to carbon dioxide, and less organic matter (total carbon, TC) remains. SS smoke is always in a free combustion state, and its combustion temperature and oxygen supply conditions are between the smoldering and open combustion of MS smoke, resulting in incomplete combustion of organic matter and the generation of a large amount of TC. In addition, the proportion of water-soluble ions (WSIIs) in PM_2.5_ in SS smoke is higher than that in MS smoke. The reason is that under the high temperature conditions of MS puffing, the three main WSIIs (NH_3_^+^, SO_4_^2−^, NO_3_^−^) will combine to form ammonium salts (such as NH_4_HSO_4_) and nitrates (NH_4_NO_3_). Under high temperature conditions, these ammonium salts and nitrates are more easily pyrolyzed into gaseous NH_3_ and HNO_3_, thereby reducing retention in the particle phase. The elemental components of PM_2.5_ in SS smoke also account for a higher proportion than MS smoke [18]. The high temperature during MS puffing will cause heavy metals (such as Cd and Pb) to volatilize [19,20], and some of them will be discharged with the airflow, while the low-temperature combustion of SS smoke makes it easier to enrich low-boiling point metals [21].

#### 3.1.2. Distribution of Major Chemical Composition in PM_2_._5_ (Carbon, Ions, and Metals)

**Figure 3:**
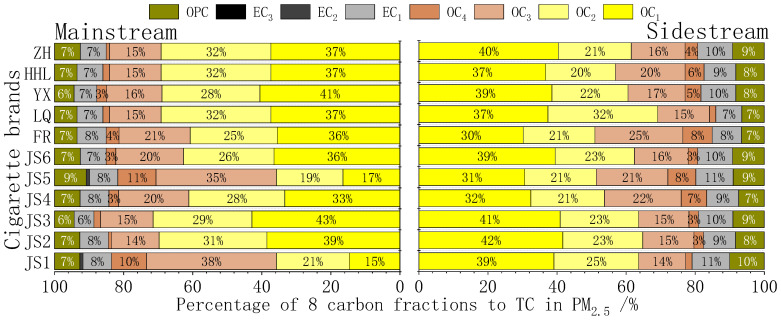
Percentage of 8 carbon fractions to TC in PM_2.5_ from main and sidestream smoke of cigarettes.

Figure 3 characterizes the distribution of eight carbon fractions in PM_2_._5_ derived from cigarette mainstream smoke (MS) and sidestream smoke (SS). Both emission types are predominantly composed of pyrolytic organic carbon fractions (OC_1_–OC_3_), collectively accounting for 81% in MS and 77% in SS. Elemental carbon is represented solely by EC_1_, contributing 7.6% in MS and 9.6% in SS. A distinct compositional divergence is observed: SS exhibits higher proportions of OC_1_ (37% vs. 34% in MS) and EC_1_ (9.6% vs. 7.6% in MS), whereas OC_2_ and OC_3_ fractions are marginally reduced. Notably, EC_2_ and EC_3_ remain either negligible or undetectable in both smoke types, suggesting minimal contribution from higher-temperature combustion products [22].

**Figure 4:**
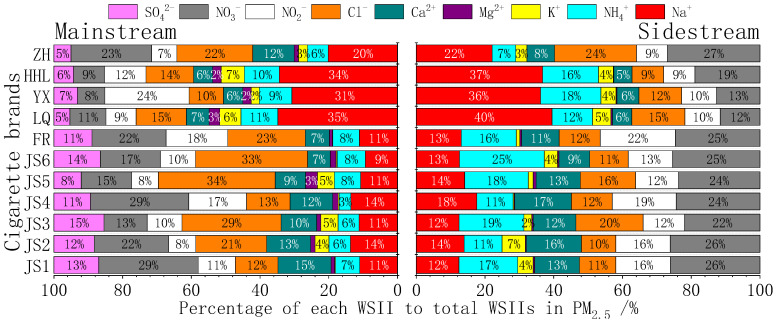
Percentage of WSIIs in PM_2.5_ from main and sidestream smoke of cigarettes.

Figure 4 ranks the distribution of nine WSIIs in PM_2.5_ across 11 cigarette brands. MS smoke is dominated by chloride (Cl^−^; 20.4%) and sulfate (SO_4_^2−^; 9.5%), whereas SS smoke exhibits significant enrichment in ammonium (NH_4_^+^; 15.4%) and nitrate (NO_3_^−^; 22.0%). Sulfate is entirely absent in SS PM_2.5_.

**Figure 5:**
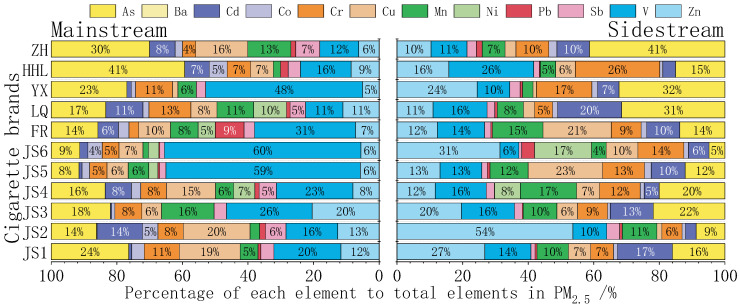
Percentage of elements in PM_2.5_ from main and sidestream smoke of cigarettes.

Figure 5 delineates the distribution of 12-element compositional profiles in PM_2.5_. MS smoke is marked by elevated vanadium (V; 29.3%) and arsenic (As; 19.4%), with cadmium (Cd; 5.5%), copper (Cu; 10.5%), and zinc (Zn; 9.3%) as secondary tracers. In contrast, SS smoke is characterized by dominant Zn (20.9%) and Cd (9.5%), followed by chromium (Cr; 11.7%) and V (13.8%). This stark contrast—V/As-dominated MS versus Zn/Cd-enriched SS—highlights the differential volatilization and condensation behaviors of elements under distinct combustion conditions [23,24].

#### 3.1.3. Toxicity-Weighted Distribution of Major PM_2.5_ Chemical Species

The carbonaceous composition reveals differential toxicity mechanisms between MS and SS. MS contains dominant OC_1_–OC_3_ fractions (81.5% of TC), indicating high concentrations of membrane-disrupting semi-volatile organics and cytochrome P450-activated PAHs/quinones [25]. SS shows elevated OC_1_ (37.0%) and EC_1_ (9.6%), enhancing oxidative stress via EC-mediated Fenton reactions and metal transport. The complete absence of EC_3_ suggests toxicity is primarily mediated through adsorbed species rather than refractory carbon matrices [26,27].

MS exhibits a Cl^−^/SO_4_^2−^ dominant profile (29.9% combined), forming acidic aerosols that induce epithelial damage and potentiate irritant effects. SS contains 37.4% NH_4_^+^/NO_3_^−^, promoting secondary inorganic aerosol formation in indoor environments [28]. Filter selectivity creates exposure dichotomy: active smokers receive higher doses of MS-derived acidic species, while passive smokers inhale SS-enriched nitrates linked to cardiopulmonary impairment [29].

In MS smoke, V is markedly enriched, averaging 29.3% (range 11.4–60.0%). At the elevated temperatures characteristic of MS combustion (800–900 °C), V-bearing species such as V_2_O_5_ and VOCl_3_ volatilize from the paper–tobacco matrix [30]. These species promptly nucleate into ultrafine particles that readily penetrate cellulose-acetate filters. Once inhaled, V catalyzes the formation of reactive oxygen species (ROS), eliciting pulmonary inflammation and oxidative DNA damage [31]. SS smoke, by contrast, is dominated by Zn, averaging 20.9% (range 10.3–53.6%). The smoldering regime (400–600 °C) and the absence of filtration jointly promote Zn release. Semi-volatile Zn species—principally ZnCl_2_ and ZnO—exhibit higher vapor pressures under these conditions; they subsequently condense onto pre-existing aerosol surfaces, resulting in downstream enrichment. Chronic exposure to elevated Zn concentrations can irritate the respiratory tract and compromise immune competence [32].

#### 3.1.4. Distribution of Ratios of OC/EC

The OC/EC ratio of particulate matter from combustion sources shows significant source dependence and can serve as an effective indicator for distinguishing different fuel types. Research indicates that the OC/EC ratio for fossil fuel combustion (coal, oil, natural gas) is typically low (<0.4), with coal combustion having the lowest ratio (0.1–0.3), reflecting its high combustion efficiency and EC dominance [33,34]. The OC/EC ratio of vehicle exhaust is slightly higher (around 0.5), with diesel vehicles potentially increasing it further to 0.4–1.2 due to incomplete combustion [35]. The range of ratios for biomass combustion is the most variable (1.5–12.0), closely related to fuel type (such as straw, wood) and combustion conditions (open burning vs. stove), with open burning leading to significant OC enrichment due to low-temperature smoldering [36].

Table 1 compares the OC and EC content in PM_2.5_ from cigarette smoke with other sources. The mean OC/EC ratio in PM_2.5_ from MS cigarette smoke was 187.3, and for SS smoke, it was 77.5. These ratios are considerably higher than those from other biomass and fossil fuel combustions, likely because cigarette and incense combustions are incomplete combustion, in contrast to the complete combustion of solid fuels, which tends to produce more EC and less OC [37].

The OC/EC ratio in PM_2.5_ from both MS and SS cigarette smoke surpasses that of Total Suspended Particulates (TSPs) from coal combustion. This difference is attributed to the size distribution of coal combustion particles, with a notable presence of coarse particles, and a decreasing OC/EC ratio as particle size increases within the range of 0.95 μm to 10 μm [16].

The OC/EC ratio reflects combustion completeness, with higher ratios indicating greater proportions of oxygenated organic carbon (OC) species. Previous experimental studies [45,46] suggest that PM_2.5_ with OC/EC > 100 is associated with a 2- to 3-fold increase in inflammatory (IL-6) and oxidative stress (8-OHdG) biomarkers in both in vitro and in vivo models. While this highlights the potential role of carbonaceous components in PM_2.5_ toxicity, the current study did not measure biological endpoints; thus, mechanistic linkages should be interpreted cautiously in the context of the prior literature.

### 3.2. Emission Factors (EFs) of PM_2.5_ and Its Chemical Composition in Smoke of Cigarettes

#### 3.2.1. Emission Factors of PM_2.5_

**Figure 6:**
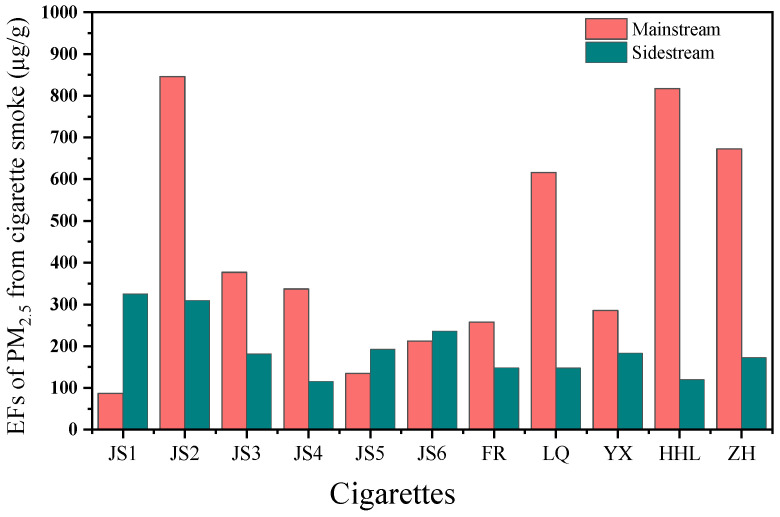
Emission factors of PM_2.5_ from main and sidestream smoke of cigarettes.

Figure 6 delineates the EFs of PM_2.5_ from MS and SS smoke across various cigarette brands and models. JS2 cigarette exhibited the highest PM_2.5_ EFs in MS smoke at 845.8 μg/g, while JS1 cigarette had the lowest at 87.1 μg/g, with other brands falling between 100 and 850 μg/g. SS smoke PM_2.5_ EFs peaked in JS1 cigarette at 325.1 μg/g and reached a minimum in JS4 cigarette at 115.6 μg/g, with other brands spanning 100 to 350 μg/g. During MS smoking, the peripheral region of the cigarette tip undergoes luminous, oxygen-rich combustion, while the interior experiences oxygen-limited smoldering pyrolysis. The SS is always in free combustion, this marked difference in oxygen availability and combustion regime is likely the primary reason why the PM_2.5_ emission factor for MS smoke exceeds that for SS smoke.

These variations stem from combustion dynamics: SS smoke undergoes oxygen-limited free combustion (400–600 °C), releasing coarse particles laden with carcinogenic polycyclic aromatic hydrocarbons (PAHs) and heavy metals (e.g., As, Cd). In contrast, MS smoke experiences brief high-temperature combustion (800–900 °C) during puffing, generating ultrafine particles (<1 μm) that deeply penetrate alveoli, carrying nicotine, formaldehyde, and reactive oxygen species (ROS) [47].

Previous study indicates that coarse particles in smoke may be effectively intercepted when passing through a filter. However, fine and ultrafine particles present a greater challenge for filtration due to their small size and high diffusivity, which allows them to easily pass through the filter’s pores and enter the smoker’s respiratory tract [48]. Ultrafine MS particles transport neurotoxic nicotine and carcinogenic aldehydes (e.g., acrolein) directly to smokers’ lungs, increasing risks of COPD and lung cancer [49]. Coarse SS particles deposit PAHs (e.g., benzo[a]pyrene) and metal(loid)s in indoor environments. These persist as thirdhand smoke, posing chronic exposure risks (e.g., developmental toxicity in children, DNA damage) [50]. SS smoke contributes >60% of indoor PM_2.5_ in smoking households. Its ROS and persistent free radicals amplify oxidative stress, correlating with asthma exacerbations and cardiovascular mortality [29,51,52].

#### 3.2.2. Emission Factors of Carbon Components

**Figure 7:**
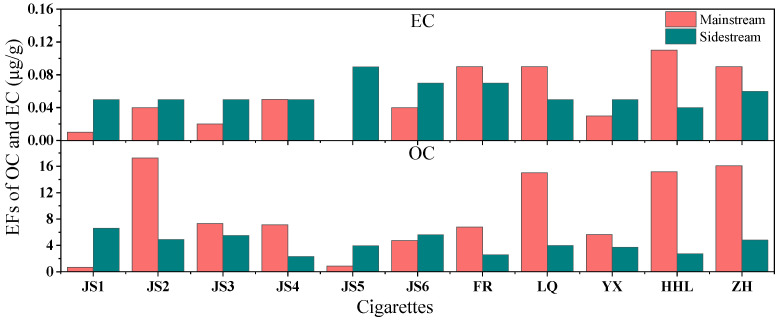
Emission factors of carbon components in PM_2.5_ from main and sidestream smoke of cigarettes.

Figure 7 highlights the variability in the EFs of OC and EC in PM_2.5_ from MS and SS cigarette smoke. The highest and lowest OC EFs in MS smoke were found in JS2 (17.3 μg/g) and JS1 (0.7 μg/g), respectively, with an average of 8.8 μg/g. For SS smoke, the OC EFs peaked in JS4 (2.35 μg/g) and troughed in JS1 (6.63 μg/g), averaging 4.3 μg/g. In terms of EC EFs, MS smoke saw the highest in HHL (0.1 μg/g) and the lowest in JS5 (not detected), averaging 0.05 μg/g. SS smoke EC EFs ranged from JS5 (0.09 μg/g) to HHL (0.04 μg/g), averaging 0.06 μg/g.

The significant variability in organic carbon (OC) and elemental carbon (EC) emission factors (EFs) between mainstream (MS) and sidestream (SS) smoke (Figure 7) presents important toxicological considerations. MS smoke exhibited substantially higher OC emissions (average 8.8 μg/g, range 0.7–17.3 μg/g) compared to SS smoke (average 4.3 μg/g, range 2.35–6.63 μg/g), suggesting that active smokers may be exposed to greater quantities of potentially harmful organic compounds. These OC components likely include polycyclic aromatic hydrocarbons (PAHs) and other carcinogenic organic species known to contribute to pulmonary and cardiovascular diseases [53,54].

Notably, the EC emissions showed an inverse pattern, with SS smoke demonstrating slightly higher average EC levels (0.06 μg/g) than MS smoke (0.05 μg/g). While absolute EC concentrations were relatively low, the presence of EC in both smoke types is concerning due to its role as a carrier for toxic compounds and its potential to induce oxidative stress in lung tissues. The detection limit challenges for EC in some samples (e.g., JS5 in MS smoke) may reflect analytical limitations rather than absence of this toxic component [55,56].

These findings highlight the differential exposure risks between active and passive smokers. The higher OC burden in MS smoke suggests greater direct toxicity for smokers, while the relatively higher EC content in SS smoke may contribute to the well-documented health risks of secondhand smoke exposure.

#### 3.2.3. Emission Factors of Water-Soluble Inorganic Ions

**Figure 8:**
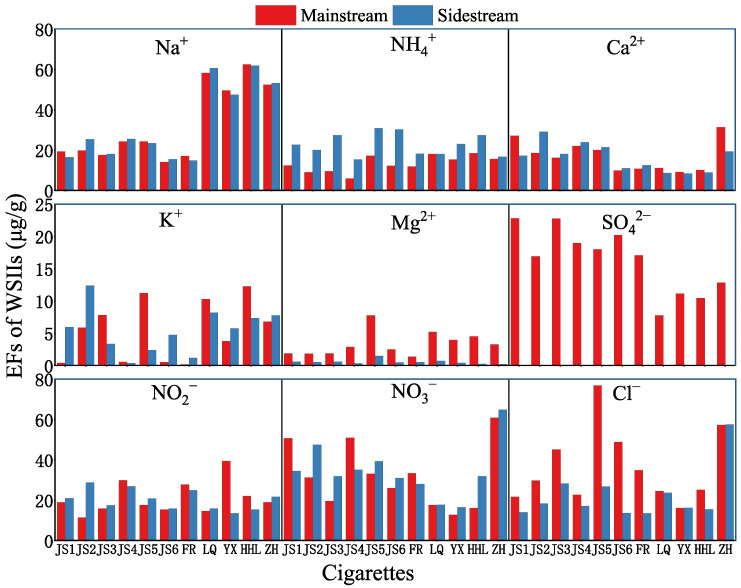
Emission factors of WSIIs in PM_2.5_ from main and sidestream smoke of cigarettes.

Figure 8 demonstrates that the EFs of Na^+^, Ca^2+^, NO_3_^−^, and NO_2_^−^ in PM_2.5_ from both MS and SS smoke across 11 cigarettes show minimal variation. In contrast, there are substantial differences in EFs of Cl^−^, K^+^, NH_4_^+^, Mg^2+^, SO_4_^2−^, and NO_2_^−^ in PM_2.5_ between from MS and SS smoke. It is noteworthy that SO_4_^2−^ was not detected in SS smoke. The reason for this is that during MS smoking, the peripheral region of the cigarette tip undergoes luminous, oxygen-rich combustion (about 800–900 °C), while the interior experiences oxygen-limited smoldering pyrolysis, the sulfides (sulfur-containing amino acids, sulfates) in tobacco will be fully oxidized to form SO_2_, and then further oxidized to SO_4_^2−^ in an oxygen-rich environment. SS smoke is produced when cigarettes are free combustion, and the combustion temperature is lower (about 400–600 °C), so the sulfide oxidation is not complete, and mainly SO_2_ is generated instead of SO_4_^2−^ [57,58].

WSIIs—especially K^+^, NH_4_^+^, and Cl^−^—are known to enhance particle hygroscopicity, leading to rapid growth of inhaled particles in the humid airway. This increases their deposition efficiency in the alveolar region and exacerbates oxidative stress and inflammatory responses [59]. The elevated Na^+^ levels in cigarette smoke further contribute to Na^+^-rich particles that may alter epithelial ion transport and mucus rheology, potentially impairing mucociliary clearance and increasing susceptibility to respiratory infections [19].

SS smoke, generated during idling periods between puffs, dominates secondhand exposure in enclosed spaces. The higher EFs of Cl^−^, K^+^, NH_4_^+^, and NO_2_^−^ in SS PM_2.5_ translate into greater indoor concentrations of these ions, raising the overall aerosol oxidative potential. This underscores the need for stringent indoor-smoking restrictions and enhanced ventilation to mitigate non-smoker exposure to these toxicologically relevant species.

#### 3.2.4. Emission Factors of Elements

**Figure 9:**
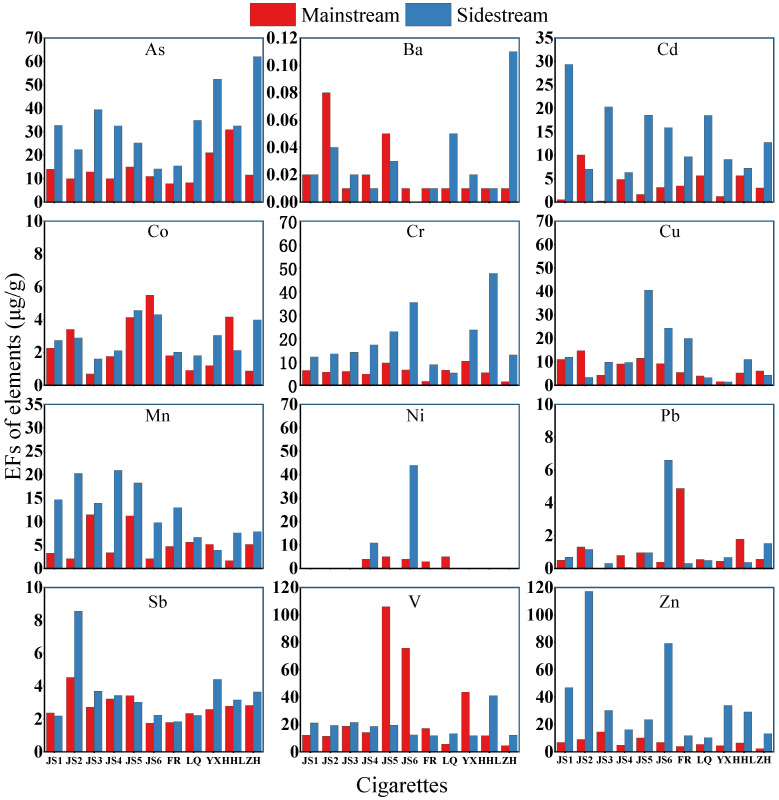
Emission factors of elements in PM_2.5_ from main and sidestream smoke of cigarettes.

Figure 9 highlights the considerable variation in EFs of V, Cr, Cu, Zn, Cd, As, and Ni in PM_2.5_ from cigarette smoke, with the difference in EFs exceeding 15 μg/g. In contrast, EFs of Mn, Co, Sb, Ba, and Pb in both MS and SS smoke showed minimal differences, some of which were below 15 μg/g. For certain cigarette models, EFs of Ba, Co, and V in MS smoke were higher than those in SS smoke, while for other elements, SS smoke had higher EFs than MS smoke, excluding Ba, Co, and V. This finding is consistent with the conclusion of previous studies [60,61,62,63] that the concentration of elements in SS smoke is generally higher than that in MS smoke. Studies have shown that cigarette filters have a dual interception effect on metal elements in MS smoke: they can effectively capture particulate metals and significantly reduce the migration of volatile metal elements [20]. Secondly, the combustion temperature causes the SS region (400–600 °C) to be more easily enriched with low-boiling point metals (such as Zn boiling point 907 °C), while elements such as V are relatively evenly distributed in the MS and SS due to their higher boiling points (3380 °C) [64,65]. The metal/metalloid elements in tobacco leaves are mainly released into the environment through the SS during combustion, while the concentration of these elements in MS smoke PM_2.5_ is low and the emission factor is small. The rest remains in the ash and filter. It is worth noting that about 15–35% of the total metal content remains in the ash and filter [4].

Cu, Zn, Cd, As, and Ni are recognized contributors to the oxidative potential of PM_2.5_. Their elevated concentrations in SS smoke enhance the ability of inhaled particles to generate reactive oxygen species (ROS) within the lung epithelium [66], promoting airway inflammation, endothelial dysfunction and, over the long term, increased cardiovascular morbidity. Cr (VI) and As (III) are additionally classified as Group 1 human carcinogens; their preferential partitioning into SS smoke therefore disproportionately elevates cancer risk for non-smokers in enclosed spaces [24,67].

Because SS smoke dominates secondhand exposure, the higher EFs of toxic metals in this fraction translate directly into greater indoor concentrations of carcinogenic and cardiopulmonary-active species. Effective mitigation requires strict source control (complete smoking bans) and, where bans are not feasible, high-efficiency ventilation or air-cleaning technologies targeting both particulate and gaseous phases.

## 4. Conclusions

This study provides quantitative emission profiles of SS smoke, establishing its significance as an indoor source of toxic PM_2.5_. Notably, SS particles exhibit 1.7–2.4-fold higher mass fractions of carcinogenic elements (As, Cd, Cr, Ni) and water-soluble ions (NH_4_^+^, Cl^−^, NO_3_^−^) compared to MS smoke. The distinctive high OC/EC ratio (mean 132) in SS smoke suggests substantial potential for oxidative stress generation. While the current study focused on emission characterization, the observed chemical profiles align with existing toxicological evidence indicating these components may contribute significantly to indoor air toxicity. These results underscore the need for further research incorporating both emission measurements and biological assessments to fully evaluate the health impacts of SS smoke exposure.

MS smoke generally exhibits higher emission factors for organic carbon (OC) and certain metals, while SS smoke demonstrates elevated levels of elemental carbon (EC) and a broader range of toxic elements and WSIIs. The distinct combustion characteristics of MS and SS smoke, driven by differences in oxygen availability and temperature, result in varying particle sizes and chemical compositions. MS smoke generates ultrafine particles that penetrate deeply into the lungs, carrying neurotoxic and carcinogenic compounds, whereas SS smoke releases coarser particles laden with carcinogens and elements that persist in indoor environments as thirdhand smoke. The higher emission factors of toxic elements and WSIIs in SS smoke underscore the disproportionate health risks posed to non-smokers through secondhand exposure.

Despite these insights, our study has limitations, including the absence of formal exposure or risk assessments to contextualize the disproportionate toxicity of cigarette aerosols. Future research should (i) expand comparative analyses to electronic and heated-tobacco products, (ii) integrate emission factors with real-world inhalation scenarios to quantify personal exposure doses, and (iii) investigate the toxicological role of enriched Zn, As, and NH_4_^+^ fractions in driving specific health endpoints. These steps are vital to inform evidence-based policies extending smoke-free regulations to all indoor microenvironments.

## Figures and Tables

**Table 1:** OC/EC in PM_2.5_ from cigarette smoke in this study and the OC/EC in particles from different other sources.

Classification of Combustibles	Combustible	Combustion State	PM Emitted from Combustion	OC/EC	Source of the Data
Cigarette	Inner layer of tobacco leaves is being smothered by the burning tip	Peripheral—luminous, oxygen-rich combustion Interior—oxygen-limited smoldering pyrolysis	PM_2.5_ from MS smoke of cigarette	187.3	This study
	Outer layer of tobacco leaves near the cigarette’s lit end	Free combustion	PM_2.5_ from SS smoke of cigarette	77.5	This study
Solid fuel	Hexagonal household coal briquet	Mixed smoldering and flaming	PM_2.5_	3.9	[38]
	Anthracite	Mixed smoldering and flaming	TSP	15.0	[39]
	Coal cakes	Mixed smoldering and flaming	TSP	8.4~14.7	[36]
	Charcoal briquette	Mixed smoldering and flaming	TSP	32.5~34.2	
	Lumber	Mixed smoldering and flaming	TSP	2.3~2.4	
	Lump coal	Smoldering	PM_1.1_	84.9	[40]
		Smoldering	PM_2.1_	81.7	
		Smoldering	PM_10_	59.4	
		Flaming	PM_1.1_	38.6	
		Flaming	PM_2.1_	37.0	
		Flaming	PM_10_	31.4	
	Manure cake	Mixed smoldering and flaming	TSP	7.9 ± 4.4	[41]
	Straw	Mixed smoldering and flaming	PM_2.5_	21.3	[42]
	Rice straw	Mixed smoldering and flaming	PM_2.5_	15.7	
	Cornstalk	Mixed smoldering and flaming	PM_2.5_	22.5	
	Foliage	Flaming	PM_2.5_ emitted during the late stage of leaf burning	2.9	[43]
		Smoldering	PM_2.5_ emitted during the initial stage of leaf burning	5.0	
Incense		Mixed smoldering and flaming	PM_2.5_	74.3	[44]

## Data Availability

All data generated or analyzed during this study are included in this published article and its Supplementary Information Files.

## Supplementary Materials

The following supporting information can be downloaded at https://www.mdpi.com/article/10.3390/toxics13090711/s1; Table S1: Parameters of cigarettes selected for experiment; Table S2: Emission factors of PM_2.5_ from main and sidestream smoke of cigarettes in this study and EFs of particles in other research; Table S3: Emission factors of water-soluble ions in PM_2.5_ from mainstream cigarette smoke (μg/g); Table S4: Emission factors of water-soluble ions in PM_2.5_ from sidestream cigarette smoke (μg/g); Table S5: Emission factors of heavy metals in PM_2.5_ from mainstream cigarette smoke(μg/g); Table S6: Emission factors of heavy metals in PM_2.5_ from sidestream cigarette smoke (μg/g). References [68,69,70,71,72,73] are cited in the supplementary materials.
