# Supplementary material for: Chemical Dissection of PM2.5 in Cigarette Smoke: Main and Sidestream Emission Factors and Compositions
Source: Toxics. 2025 Aug 23;13(9):711. doi: 10.3390/toxics13090711 (PMC12473782; doi:10.3390/toxics13090711)
Supplement: Supplementary file 1 [file toxics-13-00711-s001.zip › toxics-3744784-supplementary.pdf]

# Chemical Dissection of $PM_{2.5}$ in Cigarette Smoke: Main and Sidestream Emission Factors and Compositions


Yujian Zhou[1], Hong Huang[1], Changwei Zou[1], Mengmeng Deng[1], Xiang Tu[2], Wei Deng[2], Chenglong Yu[3], Jianlong Li[1]

[1] School of Resources & Environment, Nanchang University, Nanchang 330031, China

[2] Jiangxi Provincial Key Laboratory of Environmental Pollution Control, Jiangxi Academy of Ecological Environment Science and Planning, Nanchang 330039, China

[3] School of Land Resources and Environment, Jiangxi Agricultural University, Nanchang 330045, China


**Table S1 Parameters of cigarettes selected for experiment**

| Serial number | Cigarette brand and model | Prices ¥/pc | Weight g/pc | Weight of Filter Tip g/pc | Weight of burnable part g/pc | Length /mm | Circumference /mm | Tar mg/pc | Nicotine mg/pc | CO mg/pc |
|---|---|---|---|---|---|---|---|---|---|---|
| 1 | JS1 | 2.5 | 0.86 | 0.17 | 0.69 | 84 | 24.5 | 10 | 1 | 10 |
| 2 | JS2 | 3.5 | 0.90 | 0.32 | 0.58 | 84 | 24.5 | 10 | 1 | 10 |
| 3 | JS3 | 10 | 0.81 | 0.25 | 0.57 | 74 | 24.5 | 10 | 1 | 10 |
| 4 | JS4 | 6 | 0.67 | 0.25 | 0.42 | 88 | 24.5 | 10 | 1 | 9 |
| 5 | JS5 | 5 | 0.55 | 0.13 | 0.42 | 97 | 16 | 8 | 0.7 | 7 |
| 6 | JS6 | 9.9 | 0.93 | 0.25 | 0.68 | 84 | 24.51 | 10 | 1 | 10 |
| 7 | FR | 2.7 | 0.80 | 0.28 | 0.51 | 75 | 24.5 | 9 | 0.9 | 9 |
| 8 | LQ | 1.6 | 0.85 | 0.20 | 0.65 | 84 | 24.5 | 11 | 1 | 11 |
| 9 | YX | 2.3 | 0.91 | 0.17 | 0.74 | 84 | 24.5 | 11 | 1 | 11 |
| 10 | HHL | 3 | 0.85 | 0.29 | 0.57 | 88 | 20 | 10 | 1 | 11 |
| 11 | ZH | 4.5 | 0.90 | 0.18 | 0.72 | 84 | 24.5 | 11 | 1 | 11 |

[1] with flavor pods in the filter tip

**Table S2 Emission factors of $PM_{2.5}$ from main and sidestream smoke of cigarettes in this study and EFs of particles in other researches**

| **Classification of combustibles** | **Combustible** | **Combustion state** | **PM emissions from combustion** | **EF (μg/g)** | **Source of the data** |
|---|---|---|---|---|---|
| **Cigarette** | Inner layer of tobacco leaves is being smothered by the burning tip | Alternating between Smoldering and bright combustion within the cigarette's tail end burns the inner layer of tobacco leaves | $PM_{2.5}$ from mainstream smoke | 422.1 | This study |
| | Outer layer of tobacco leaves near the cigarette's lit end | free combustion | $PM_{2.5}$ from sidestream smoke | 193.8 | This study |
| **Fuel in residential areas** | Refuse incineration | Mixed smoldering and flaming | $PM_{2.5}$ | 7810 | (U.S. Environmental Protection Agency 2024) |
| | Anthracite | Mixed smoldering and | $PM_{2.5}$ | 1054 | (Zhang et al.,2008) |

| | | | | | |
|---|---|---|---|---|---|
| | | flaming | | | |
| | Bituminous coal | Mixed smoldering and flaming | $PM_{2.5}$ | 7373 | |
| | Briquettes | Mixed smoldering and flaming | $PM_{2.5}$ | 5242 | |
| | Peat | Mixed smoldering and flaming | $PM_{2.5}$ | 7020 | (Christian et al.,2003) |
| | Liquefied petroleum gas | Mixed smoldering and flaming | TSP | 520 | (Zhang et al.,2000) |
| | Dry natural gas | Mixed smoldering and flaming | TSP | 150 | |
| | Landfill gas | Mixed smoldering and flaming | TSP | 150 | |
| | Liquefied natural gas | Mixed smoldering and flaming | $PM_{2.5}$ | 200 | (Habib et al.,2008) |
| | Gasoline | Mixed smoldering and flaming | $PM_{2.5}$ | 700 | |
| | Manure cake | Mixed smoldering and flaming | $PM_{2.5}$ | 5400 | |
| **Crop residue** | Straw | Mixed smoldering and flaming | $PM_{2.5}$ | 11400 | (Ni et al.,2017) |
| | Rice straw | Mixed smoldering and flaming | $PM_{2.5}$ | 8500 | |
| | Cornstalk | Mixed smoldering and flaming | $PM_{2.5}$ | 12000 | |
| **Branches of tree** | | Flaming | $PM_{2.5}$ | 17400 | (Reisen et al.,2018) |
| | | Smoldering | $PM_{2.5}$ | 49800 | |

**Table S3 Emission factors of water-soluble ions in $PM_{2.5}$ from mainstream cigarette smoke(μg/g)**

| Ions / Cigarettes | $Na^+$ | $NH_4^+$ | $K^+$ | $Mg^{2+}$ | $Ca^{2+}$ | $NO_2^-$ | $NO_3^-$ | $SO_4^{2-}$ |
|---|---|---|---|---|---|---|---|---|
| JS1 | 19.24 | 12.30 | 0.41 | 1.89 | 27.06 | 18.95 | 50.68 | 22.80 |
| JS2 | 19.79 | 8.97 | 5.84 | 1.85 | 18.51 | 11.45 | 31.19 | 16.91 |
| JS3 | 17.49 | 9.55 | 7.82 | 1.86 | 16.22 | 15.83 | 19.64 | 22.76 |
| JS4 | 24.18 | 5.96 | 0.58 | 2.88 | 22.04 | 29.89 | 50.93 | 18.99 |
| JS5 | 24.21 | 17.24 | 11.18 | 7.74 | 20.02 | 17.62 | 32.99 | 17.98 |
| JS6 | 14.00 | 12.22 | 0.49 | 2.51 | 9.86 | 15.29 | 26.04 | 20.20 |
| FR | 17.10 | 11.95 | 0.19 | 1.39 | 10.81 | 27.72 | 33.32 | 17.09 |
| LQ | 58.21 | 17.97 | 10.29 | 5.22 | 11.07 | 14.64 | 17.57 | 7.74 |
| YX | 49.53 | 15.25 | 3.76 | 3.97 | 9.09 | 39.43 | 12.77 | 11.13 |
| HHL | 62.44 | 18.47 | 12.26 | 4.51 | 10.11 | 22.06 | 16.18 | 10.42 |
| ZH | 52.28 | 15.62 | 6.79 | 3.27 | 31.24 | 18.98 | 60.84 | 12.86 |

**Table S4 Emission factors of water-soluble ions in $PM_{2.5}$ from sidestream cigarette smoke(μg/g)**

| Ions / Cigarettes | $Na^+$ | $NH_4^+$ | $K^+$ | $Mg^{2+}$ | $Ca^{2+}$ | $NO_2^-$ | $NO_3^-$ | $SO_4^{2-}$ |
|---|---|---|---|---|---|---|---|---|
| JS1 | 16.41 | 22.59 | 5.94 | 0.59 | 17.24 | 13.97 | 21.01 | 34.44 |
| JS2 | 25.28 | 20.03 | 12.35 | 0.54 | 29.05 | 18.22 | 28.79 | 47.39 |
| JS3 | 18.01 | 27.33 | 3.35 | 0.62 | 17.99 | 28.31 | 17.48 | 31.74 |
| JS4 | 25.37 | 15.24 | 0.35 | 0.31 | 23.82 | 17.13 | 26.89 | 35.08 |
| JS5 | 23.41 | 30.77 | 2.36 | 1.48 | 21.39 | 26.71 | 20.79 | 39.37 |

| | | | | | | | | |
|---|---|---|---|---|---|---|---|---|
| JS6 | 15.36 | 30.13 | 4.74 | 0.47 | 10.94 | 13.68 | 15.77 | 31.04 |
| FR | 14.85 | 18.19 | 1.18 | 0.55 | 12.42 | 13.53 | 24.88 | 27.95 |
| LQ | 60.57 | 18.05 | 8.20 | 0.70 | 8.60 | 23.71 | 15.81 | 17.67 |
| YX | 47.45 | 23.03 | 5.73 | 0.38 | 8.35 | 16.27 | 13.43 | 16.51 |
| HHL | 61.85 | 27.32 | 7.32 | 0.24 | 8.85 | 15.49 | 15.30 | 31.89 |
| ZH | 53.14 | 16.57 | 7.74 | 0.22 | 19.30 | 57.41 | 21.70 | 64.79 |

**Table S5 Emission factors of Heavy metals in $PM_{2.5}$ from mainstream cigarette smoke(μg/g)**

| Ions / Cigarettes | V | Cr | Mn | Co | Ni | Cu | Zn | As | Cd | Sb | Ba | Pb |
|---|---|---|---|---|---|---|---|---|---|---|---|---|
| JS1 | 12.17 | 6.36 | 3.25 | 2.26 | 0.00 | 11.08 | 6.94 | 14.09 | 0.52 | 2.37 | 0.02 | 0.50 |
| JS2 | 11.46 | 5.61 | 2.11 | 3.41 | 0.00 | 14.79 | 9.15 | 10.03 | 10.07 | 4.54 | 0.08 | 1.32 |
| JS3 | 18.75 | 5.90 | 11.47 | 0.69 | 0.00 | 4.32 | 14.61 | 12.91 | 0.27 | 2.72 | 0.01 | 0.00 |
| JS4 | 14.20 | 4.75 | 3.36 | 1.75 | 3.96 | 9.11 | 4.86 | 10.01 | 4.84 | 3.22 | 0.02 | 0.79 |
| JS5 | 105.99 | 9.48 | 11.22 | 4.14 | 4.98 | 11.52 | 10.21 | 15.06 | 1.63 | 3.42 | 0.05 | 0.97 |
| JS6 | 75.68 | 6.49 | 2.11 | 5.49 | 4.01 | 9.21 | 6.99 | 10.95 | 3.15 | 1.75 | 0.01 | 0.39 |
| FR | 17.14 | 1.63 | 4.68 | 1.80 | 2.93 | 5.43 | 4.02 | 7.91 | 3.48 | 1.79 | 0.01 | 4.87 |
| LQ | 5.69 | 6.40 | 5.60 | 0.91 | 5.01 | 3.97 | 5.53 | 8.32 | 5.65 | 2.35 | 0.01 | 0.55 |
| YX | 43.67 | 10.31 | 5.14 | 1.19 | 0.00 | 1.49 | 4.60 | 21.04 | 1.23 | 2.59 | 0.01 | 0.44 |
| HHL | 11.75 | 5.41 | 1.66 | 4.16 | 0.00 | 5.27 | 6.46 | 30.86 | 5.65 | 2.79 | 0.01 | 1.79 |
| ZH | 4.60 | 1.57 | 5.10 | 0.87 | 0.00 | 6.14 | 2.42 | 11.59 | 3.02 | 2.83 | 0.01 | 0.57 |

**Table S6 Emission factors of Heavy metals in $PM_{2.5}$ from sidestream cigarette smoke(μg/g)**

| Ions / Cigarettes | V | Cr | Mn | Co | Ni | Cu | Zn | As | Cd | Sb | Ba | Pb |
|---|---|---|---|---|---|---|---|---|---|---|---|---|
| JS1 | 24.67 | 12.10 | 16.79 | 2.18 | 0.00 | 11.97 | 46.89 | 28.02 | 29.37 | 2.19 | 0.02 | 1.03 |
| JS2 | 22.55 | 13.40 | 23.15 | 2.31 | 0.00 | 3.33 | 117.12 | 19.13 | 7.04 | 8.56 | 0.04 | 1.73 |
| JS3 | 25.03 | 14.09 | 15.92 | 1.28 | 0.00 | 9.79 | 30.33 | 33.76 | 20.30 | 3.69 | 0.02 | 0.45 |
| JS4 | 21.62 | 17.26 | 23.87 | 1.69 | 10.92 | 9.77 | 16.22 | 27.89 | 6.36 | 3.44 | 0.01 | 0.10 |
| JS5 | 22.71 | 22.88 | 20.84 | 3.65 | 0.00 | 40.59 | 23.46 | 21.68 | 18.54 | 3.03 | 0.03 | 1.45 |
| JS6 | 14.49 | 35.39 | 11.16 | 3.44 | 43.92 | 24.37 | 79.07 | 12.18 | 15.89 | 2.24 | 0.00 | 9.89 |
| FR | 13.76 | 8.76 | 14.82 | 1.61 | 0.00 | 19.99 | 11.84 | 13.21 | 9.69 | 1.84 | 0.01 | 0.46 |
| LQ | 15.59 | 5.21 | 7.57 | 1.44 | 0.00 | 3.23 | 10.43 | 29.84 | 18.50 | 2.22 | 0.05 | 0.73 |
| YX | 13.82 | 23.57 | 4.46 | 2.43 | 0.00 | 1.41 | 34.04 | 44.97 | 9.12 | 4.41 | 0.02 | 1.01 |
| HHL | 47.99 | 47.75 | 8.69 | 1.70 | 0.00 | 11.03 | 29.18 | 27.82 | 7.22 | 3.16 | 0.01 | 0.55 |
| ZH | 14.17 | 12.90 | 8.99 | 3.19 | 0.00 | 4.29 | 13.31 | 53.15 | 12.71 | 3.65 | 0.11 | 2.29 |


1. Christian, T.J., Kleiss, B., &Yokelson, R.J. (2003). Comprehensive laboratory meas urements of biomass-burning emissions: Emissions from Indonesian, African, and o ther fuels. J. Geophys. Res. 108:(D23). https://doi.org/10.1029/2003JD003704
2. Habib, G., Venkataraman, C., Bond, T.C., &Schauer, J.J. (2008). Microphysical and Optical

Properties of Primary Particles from the Combustion of Biomass Fuels. Environ. Sci. Technol. 42(23):8829-8834. https://doi.org/10.1021/es800943f

3. Ni, H., Tian, J., &Wang, X. (2017). $PM_{2.5}$ Emissions and Source Profiles from Open Burning of Crop Residues. China, Atmos. Environ.169:229-237. https://doi.org/10.1016/j.atmosenv.2017.08.063
4. Reisen, F., Meyer, C.P., Weston, C.J., &Volkova, L. (2018). Ground-Based Field Measurements of $PM_{2.5}$ Emission Factors From bright combustion and smoldering combustion in Eucalypt Forests. J. Geophys. Res. 123:8301-8314. https://doi.org/10.1029/2018JD028488
5. U.S. Environmental Protection Agency. Clearinghouse for Inventories & Emissions factors, http://www.epa.gov/ttn/chief/index.html.
6. Xu, H., Li, Y., &Guinot, B. (2018). Household Heating and Cooking in Rural Guanzhong Plain, Northwestern China, Atmos. Environ. 185:196-206.
7. Zhang, Y., Schauer, J., &Zhang, Y. (2008). Characteristics of particulate carbon emissions from real-world Chinese coal combustion. China, Environ. Sci. Technol. 42(14):5068-5073. https://doi.org/10.1021/acs.est.7b01543
8. Zhang, J., Smith, K.R., &Ma, Y. (2000). Greenhouse gases and other airborne pollutants from household stoves in China: a database for emission factors. China, Atmos. Environ. 34(26):4537-4549. https://doi.org/10.1016/S1352-2310(99)00450-1
